# Triboelectric Nanogenerators for Vehicle Energy Harvesting and Intelligent Sensing

**DOI:** 10.3390/mi17080975

**Published:** 2026-08-18

**Authors:** Chuanqing Zhu, Yatong Ren, Ziyue Xi, Hengxu Du

**Affiliations:** 1 School of Mechanical and Automotive Engineering, Liaocheng University, Liaocheng 252000, China; renyatong2026@163.com; 2 Marine Engineering College, Dalian Maritime University, Dalian 116026, China; yyds@dlmu.edu.cn (Z.X.); duhengxu@foxmail.com (H.D.)

**Keywords:** triboelectric nanogenerator (TENG), energy harvesting, intelligent sensing, vehicle applications

## Abstract

As vehicle intelligence and automotive electrification advance, the extensive deployment of distributed sensing nodes for comprehensive monitoring has grown rapidly. This poses severe challenges, such as rising onboard power consumption and the inability of conventional centralized power supply systems to sustain these sensors. Triboelectric nanogenerators (TENGs), an emerging technology for energy harvesting and self-powered sensing, exhibit great potential to address the above challenges. This review systematically summarizes research on TENGs for vehicle energy harvesting and intelligent sensing, covering their fundamental working principles and applications in diverse vehicle scenarios. First, the basic principle and working modes of TENGs are described, and their suitability for complex and variable vehicle environments is evaluated. Subsequently, existing applications are categorized into three domains: vehicle vibration systems, wheel–road systems, and intelligent vehicle systems. Studies on various topics are reviewed, including vibration energy harvesting and sensing, vehicle collision monitoring, tire energy harvesting, road sensing, smart cockpits, human–machine interaction, and vehicle fluid monitoring. Emphasis is placed on their technical approaches and application prospects. Finally, the state-of-the-art research and prevailing technical bottlenecks are summarized, and potential solutions and future research perspectives are discussed. This review aims to support the reliable practical deployment of TENG technology in vehicle engineering and to provide a technical basis for energy-saving strategies and in situ sensing technologies for future intelligent vehicles.

## 1. Introduction

Vehicle electrification [1,2], connectivity, and intelligence [3,4] are advancing in parallel, reshaping the modern vehicle from a conventional electromechanical control system into an intelligent mobile platform. High-precision environmental perception [5], multisource information processing [6,7], wireless communication, and active vehicle control provide the functional basis for this transition [8,9,10,11]. Progress toward higher levels of driving automation and connected-vehicle operation has consequently increased demand for onboard multimodal sensors, high-performance computing platforms, and electronic actuators. Lidar, millimeter-wave radar, ultrasonic sensors, and high-resolution cameras are among the principal onboard sensing technologies [12,13]. Broader deployment of these components has improved perception accuracy and real-time decision-making capabilities under complex traffic scenarios [14,15,16].

These gains in vehicle intelligence and vehicle-wide condition monitoring, however, inevitably add vehicle mass, aerodynamic drag, and dynamic power consumption, thereby reducing usable driving range [17,18]. Many key monitoring nodes must also operate in harsh locations exposed to severe impacts or subject to stringent sealing requirements. Conventional wired connections at these locations remain vulnerable to fatigue-induced failure during prolonged service. The rapid growth in distributed sensor nodes further increases wiring-harness complexity and, consequently, installation and maintenance costs. Such penalties conflict directly with lightweight vehicle design and low-cost maintenance requirements [19]. A central engineering challenge is therefore to provide distributed low-power sensor nodes with reliable long-term power without appreciably increasing system size or maintenance burden [20,21,22,23]. Resolving this challenge is important for the large-scale deployment and reliable operation of intelligent connected vehicles.

During vehicle operation, only part of the power produced by the engine or electric motor is used to overcome running resistance. A substantial portion is dissipated without recovery through high- and low-frequency vehicle body vibrations, reciprocating suspension motion, cyclic tire deformation, and dynamic wheel–road friction [24,25,26]. Previous studies have identified considerable energy recovery potential in several key vehicle components, particularly suspension systems. The recoverable energy is strongly affected by road roughness class, vehicle speed, spring stiffness, damping characteristics, and other operating conditions [27,28]. These results support both the feasibility and practical value of recovering distributed mechanical energy that would otherwise be lost in vehicles [29]. The main limitation, however, is the ability of available energy conversion technologies to accommodate these spatially dispersed energy sources. Current approaches to onboard mechanical energy harvesting rely primarily on electromagnetic generators (EMGs) [30] and piezoelectric nanogenerators (PENGs) [31,32,33]. EMGs generate electricity from relative motion between a magnet and a coil, but their output depends strongly on structural resonance and high-frequency motion. Their comparatively bulky and heavy architectures also exhibit sharply reduced conversion efficiency under low-frequency or irregular mechanical excitation [34]. PENGs provide more compact structures, yet their energy output relies heavily on localized high-strain deformation of piezoelectric materials. Large displacements, strong impacts, and broadband random vibrations in vehicles can therefore severely limit their mechanical durability and output stability [35,36]. Vehicle environments consequently require an energy conversion and electromechanical conversion technology capable of responding to wideband, nonstationary, and nonlinear excitations from multiple sources. Such a technology should combine low mass and mechanical compliance with effective operation across a broad range of vehicle conditions.

The triboelectric nanogenerator (TENG) was first reported by Wang et al. [37] in 2012 based on the combined effect of contact electrification and electrostatic induction. TENGs can efficiently convert even small amounts of mechanical energy into electrical power via the use of contact-separation, sliding, rolling, single-electrode, and freestanding triboelectric-layer modes. Their main characteristics are a very large variety of materials, great flexibility in structural design, low weight, and good adaptability to low-frequency excitations in a wide amplitude range [38,39]. This was followed by subsequent theoretical work and then the establishment of a systematic distributed-parameter model for electromechanical coupling [40]. This was followed by a comprehensive figure of merit for the evaluation of the performance of different TENG materials and configurations [41]. In terms of energy conversion, the output of TENG can be explained by Maxwell’s displacement current theory [42] and the electron-transfer process of contact electrification [43]. The output magnitude is also related to the triboelectric properties of the constituent materials [44] and the interfacial contact electrification mechanisms [45]. The open-circuit voltage, short-circuit current, and transferred charge show strong and systematic dependence on excitation amplitude, frequency, force direction, and contact condition for the mechanical-to-electrical transduction process. This electromechanical correspondence allows a TENG to operate as both an energy harvester [46,47] and a self-powered sensor [48,49,50,51,52]. The resulting coupling between energy conversion and information transduction [53,54] has motivated studies of wheel rotation and wheel deformation energy recovery [55] and multidirectional vehicle vibration harvesting [56]. Reported sensing applications include vehicle motion state monitoring and collision warning [57], vector motion monitoring in automated vehicles [58], and real-time monitoring of traffic flow and road conditions [59]. TENGs have additionally been applied to smart cockpit interaction [60] and to detecting driver fatigue and distraction [61]. Collectively, these demonstrations support the use of TENGs in distributed onboard self-powered sensing networks that can operate without batteries and require no maintenance [62].

Previous reviews have addressed TENG applications in conventional vehicle systems and in specific transportation scenarios, but a systematic account of TENG-based energy harvesting and intelligent sensing across the integrated human–vehicle–road system is still lacking. This review therefore first summarizes the fundamental mechanisms of TENGs and their suitability for vehicle use and progresses from the vehicle body structures and wheel–road systems to intelligent in-vehicle interactions, systematically reviewing recent innovations and technological advances in TENG-based vehicle energy harvesting and intelligent sensing. Representative applications are analyzed in terms of their technical methodologies and engineering potential. These applications include vehicle vibration energy harvesting, collision monitoring and alert systems, wheel mechanical energy harvesting, intelligent road sensing, human–machine interaction in smart cockpits, and vehicle fluid monitoring. The evaluation concludes with a discussion of the major challenges to large-scale vehicle application, including material durability, ecological adaptability, and multi-source signal processing, as well as potential directions for future development. This review aims to provide a comprehensive theoretical reference and practical engineering guidance for the implementation and deployment of TENGs in intelligent connected vehicles.

## 2. TENG Fundamentals and Vehicle Suitability

### 2.1. Basic Principle and Fundamental Working Models

The fundamental operating principle of a triboelectric nanogenerator arises from the coupled effects of contact electrification and electrostatic induction and is theoretically de-scribed by Maxwell’s displacement current theory [42,43]. Under periodic external mechanical excitation, different dielectric materials repeatedly come into contact and separate. During contact, electrostatic charges are generated and accumulated on the contacting surfaces through contact electrification. Once surface charges form on the dielectric materials, equal and opposite charges are induced on the corresponding electrodes to balance the resulting potential difference. This phenomenon is known as electrostatic induction. Periodic charge generation is due to repetitive contact–separation cycles. Connecting the electrodes via an external load drives bidirectional charge transfer in the circuit, which produces an alternating current. In this way, the TENG achieves effective and continuous conversion of mechanical energy into electrical energy. The electrical output signals generated in this process show a clear dynamic correspondence with the applied mechanical excitation, providing the physical basis for the device’s remarkable self-powered sensing abilities. In particular, the output voltage, current, and transferred charge show different responses to variation of excitation amplitude, motion frequency, and contact area. This direct correlation between mechanical stimulation and electrical signal enables the TENG to be a self-powered sensor without the need for an external power source.

**Figure 1:**
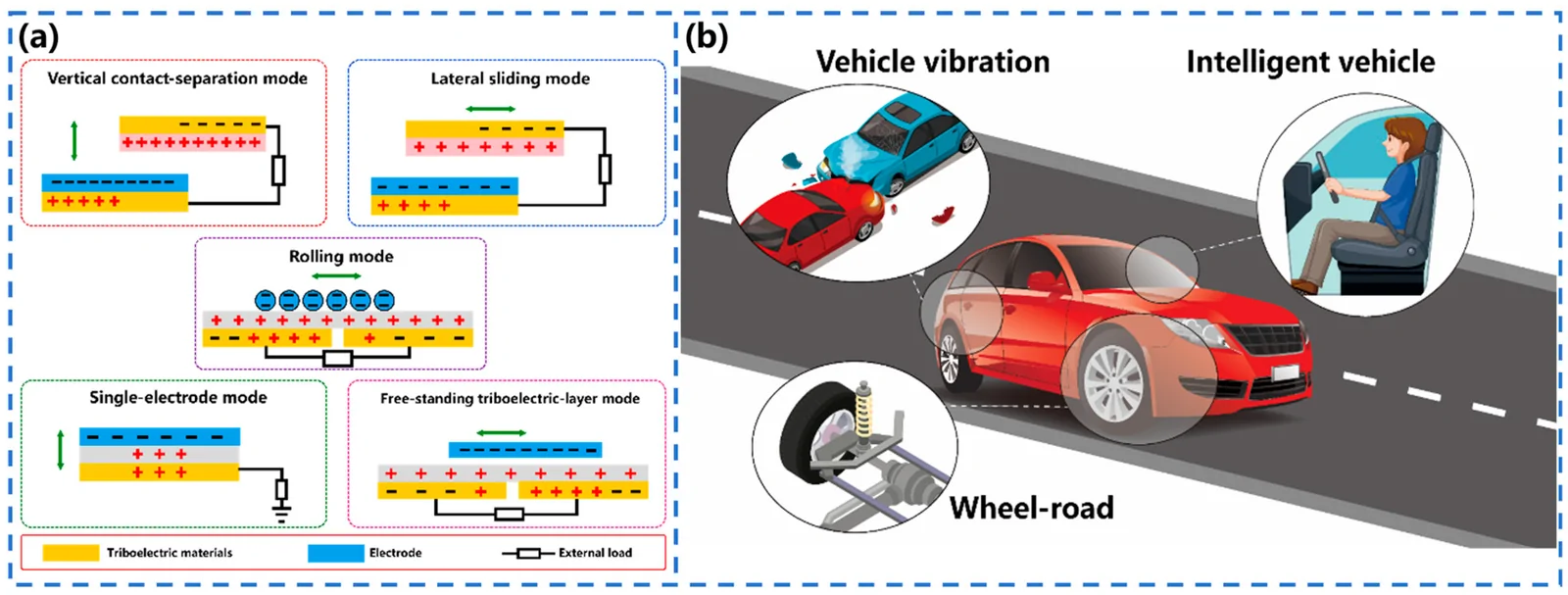
Fundamental TENG operating modes and representative vehicle application scenarios. (**a**) Vertical contact–separation, lateral sliding, rolling, single-electrode, and freestanding triboelectric-layer modes. (**b**) Representative application areas involving vehicle vibration, wheel–road interaction, and smart cockpits.

TENGs can be grouped into five fundamental operating modes according to the applied mechanical excitation, as illustrated in Figure 1a: vertical contact–separation, lateral sliding, single-electrode, freestanding triboelectric-layer, and rolling modes. Combined with the wide selection of triboelectric materials, these operating modes provide substantial freedom in structural design and support diverse TENG applications.

### 2.2. Suitability for Vehicle Applications

During vehicle operation, considerable mechanical energy is dissipated unrecovered through vibration, friction, and related processes [24]. Recovering part of this energy could extend driving range and support further development of vehicle intelligence. Conventional generators, however, face substantial difficulty harvesting such high-entropy mechanical energy because the available excitations are largely low in frequency and amplitude and are highly stochastic [63].

Traditional electromagnetic generators (EMGs) are constrained by the mass of their coils and magnets and therefore often have difficulty achieving sufficient relative motion under low vibration acceleration [64]. Because the voltage induced by EMGs depends strongly on excitation frequency and the number of turns of the coil, miniaturized devices exposed to typical vehicle vibrations generally produce exceptionally low voltage output that frequently fails to reach the start-up threshold of general commercially available power-management modules (typically above 2.5 V). Their numerous rigid moving components are also prone to mechanical wear under harsh vehicle vibration and make thin, compact integration difficult in space-limited locations. Piezoelectric nanogenerators (PENGs), by comparison, offer lightweight structures and relatively broad frequency adaptability, but their electromechanical conversion depends strictly on deformation of piezoelectric crystals [65,66]. The low-acceleration excitation encountered during routine driving often makes it difficult to induce sufficient and effective crystal deformation, thereby restricting electrical output [32,67].

TENGs, by contrast, are well suited to harvesting high-entropy mechanical energy. Their electromechanical conversion is governed by contact electrification and relative displacement between materials rather than by bulk crystal deformation. This mechanism substantially reduces the acceleration threshold required for effective operation. Reported studies show that TENGs can harvest vibration over a broad frequency range of 0–250 Hz [68] while remaining responsive at low acceleration levels of 5 m/s^2^ [69].

Many vehicle components experience stochastic, low-amplitude vibration within the effective energy-harvesting range of TENGs, while periodic motions such as suspension reciprocation and wheel rotation provide comparatively stable excitation. TENGs also inherently support self-powered sensing [70]. When coupled with suitable impedance-matching and energy management circuits, their electrical output has strong potential to supply part of the in situ power demand of distributed low-power vehicle sensors and thereby reduce dependence on external wiring. Under optimized conditions, TENG-based sensors can provide competitive sensitivity and may enable extended operational lifespans or quasi-maintenance-free monitoring. These combined energy-harvesting and sensing characteristics make TENGs promising for vehicle vibration recovery, vehicle–road interaction, and smart cockpit applications, as summarized in Figure 1b.

## 3. Vehicle Vibration Systems

Vehicle operation generates significant vibrational energy from constant engine excitation, suspension oscillations, and tire interactions with irregular road surfaces. Much of this mechanical energy is typically wasted as heat instead of being used. This leads to considerable resource inefficiency. Efficiently recovering this otherwise lost vibrational energy and converting it into a long-lasting, environmentally benign source of electricity could enable the self-powering of onboard microelectronic devices.

### 3.1. Vehicle Vibration

Among automotive vibration sources, structural vibration of the vehicle is among the most widely distributed and offers substantial potential for energy recovery and reuse. Beyond the high-frequency vibration produced by the engine, random road excitation induces passive vibration throughout the chassis, cabin frame, and internal components under varying road conditions [25]. Because TENGs respond effectively to low-frequency, high-entropy mechanical inputs, they are well suited to harvesting energy from these distributed vibration sources across different vehicle operating conditions.

**Figure 2:**
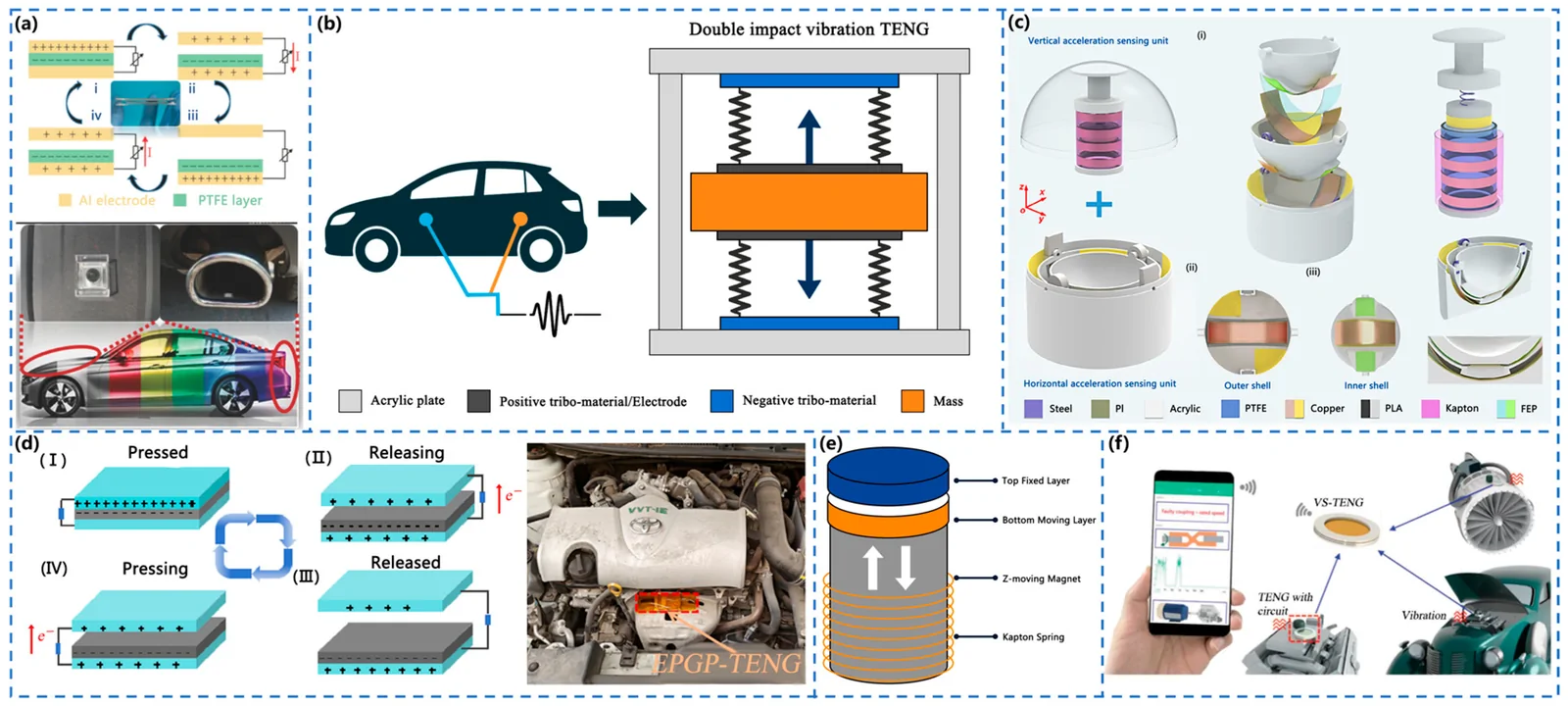
Triboelectric energy harvesting and self-powered monitoring of vehicle body and powertrain vibrations. (**a**) Operating principle and installation location of the EV-TENG [71]. (**b**) Double-impact vibration TENG (DIV-TENG) adapted from [72]. (**c**) Gyroscope-inspired triboelectric three-axis acceleration sensor (GTTAS) [73]. (**d**) Ex-Pe@G–PDMS foam TENG (EPGP-TENG) for abnormal engine vibration monitoring [75]. (**e**) Self-powered triboelectric vibration sensor (SP-TVS) integrated with a vehicle energy-harvesting module (VEHM) and the corresponding onboard test setup adapted from [76]. (**f**) Structure of the triboelectric vibration sensor (VS-TENG) and its application to mechanical equipment condition monitoring [77].

Shen et al. [71] developed an engine vibration energy-harvesting TENG (EV-TENG), with its operating principle and installation location illustrated in Figure 2a. A heavy iron block mounted on top of the TENG increased contact–separation motion between the upper and lower sections, and its mass could be adjusted to accommodate different engine types. Insulating sheets placed beneath the device protected it from engine heat, while the harvested vibration energy could power a gas monitoring sensor. For a wider vibration range, Kim et al. [72] investigated a double-impact vibration TENG (DIV-TENG) for automotive energy harvesting. Figure 2b shows eight springs divided into two stiffness groups, allowing effective response at both low and high frequencies. Beyond energy harvesting, sensing and monitoring vehicle body vibration are equally important because the vibration contains information on operating conditions in addition to recoverable mechanical energy. To resolve vibration along multiple degrees of freedom, Yang et al. [73] proposed a gyroscope-inspired triboelectric three-axis acceleration sensor (GTTAS). The soft-contact and noncontact electrode configurations shown in Figure 2c enabled operation at low accelerations. The mass block could vibrate freely within the PTFE tube as the FEP repeatedly contacted the forked copper electrode, allowing measurement of vertical and horizontal vehicle acceleration. For the high-temperature vibration environment around an engine, Han et al. [74] developed a high-temperature sensing and energy-harvesting TENG resembling a conventional piezoelectric knock sensor. Cordierite (Crd; 2MgO·2Al_2_O_3_·5SiO_2_) was incorporated into a polyimide (PI) matrix, and the resulting Crd–PI composite retained triboelectric charge at elevated temperature. Wang et al. [75] prepared expanded perlite@graphene (Ex-Pe@G) by a simple carbonization reaction and dispersed it in polydimethylsiloxane (PDMS) to produce porous Ex-Pe@G–PDMS foam (EPGP). As shown in Figure 2d, this structure could function as a self-powered high-temperature sensor. To extend both dimensional and frequency coverage, Bhatta et al. [76] designed the highly sensitive self-powered triboelectric vibration sensor (SP-TVS) shown in Figure 2e. The device monitored impact and vibration over a range of amplitudes associated with vehicle motion on different road surfaces. It exhibited excellent frequency- and acceleration-response characteristics, and the output voltage increased approximately linearly with acceleration. Zhao et al. [77] further broadened the monitored frequency range using an aluminum foam/FEP film TENG, as shown in Figure 2f. These studies demonstrate the applicability of TENGs to both vehicle body vibration energy harvesting and vibration sensing, while integrating the two functions into passive intelligent sensing remains an important technical challenge.

### 3.2. Suspension Vibration

The suspension system forms the principal mechanical link between the vehicle body and the wheels and attenuates road-induced disturbances to maintain ride comfort and handling stability. During operation, however, uneven road surfaces impose variable-frequency and variable-stroke excitation that repeatedly extends and compresses the suspension. The resulting vibration is dominated by low-frequency, large-stroke motion with strongly dynamic and stochastic characteristics, closely matching the operating regime of TENGs. Multiple TENG working modes can therefore harvest weak, low-frequency suspension vibration without altering or adversely affecting the original suspension dynamics.

**Figure 3:**
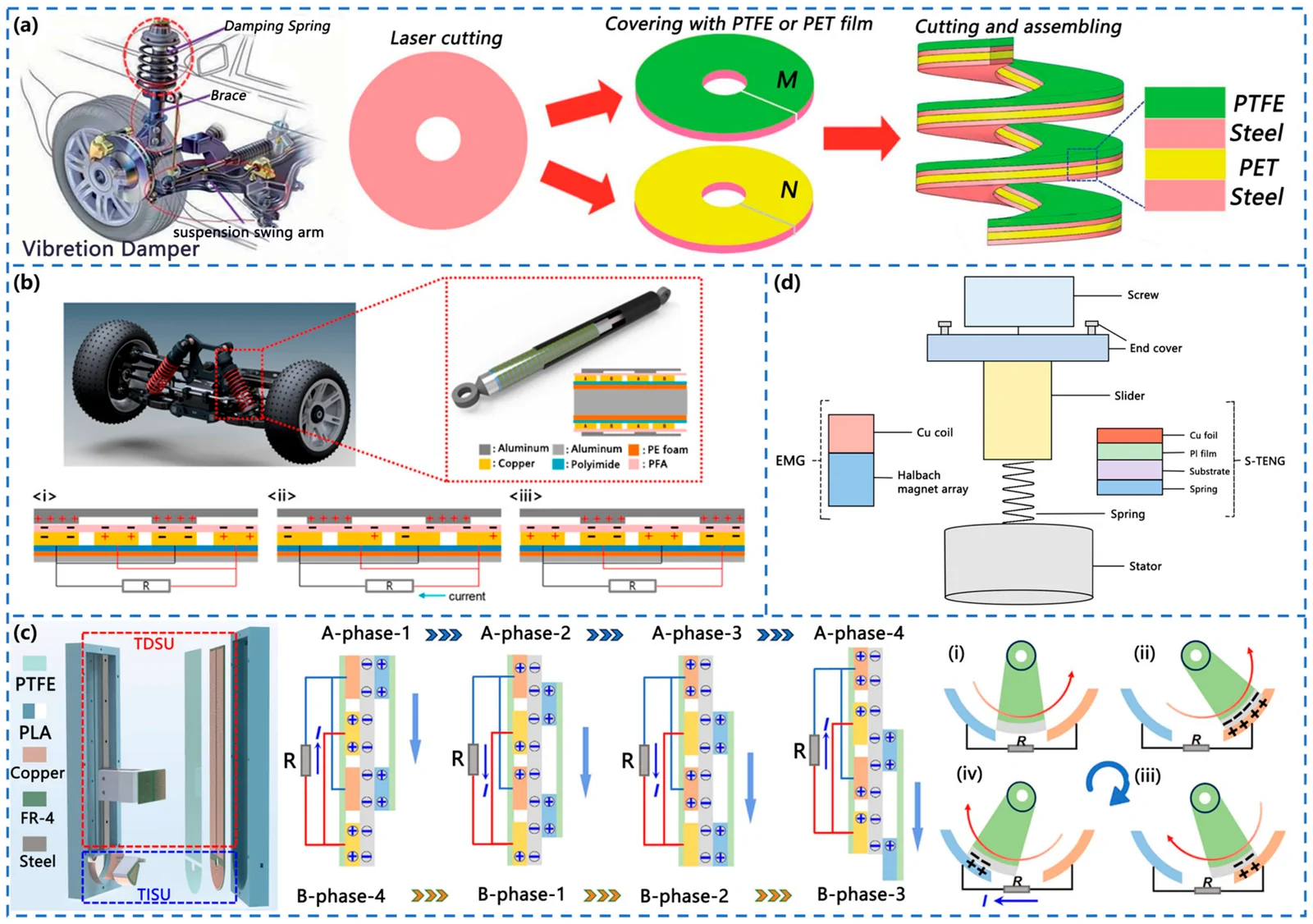
Triboelectric vibration energy harvesting and road condition sensing in automotive suspension systems. (**a**) Suspension installation, fabrication, and structure of the retractable spring-like-electrode TENG (SL-TENG) [78]. (**b**) Integration and operating process of the cylindrical freestanding-mode triboelectric generator (STEG) in a shock absorber [79]. (**c**) Structure of the triboelectric dual-parameter sensor (TDS) and its displacement- and inclination-sensing principles [80]. (**d**) Embedded TENG–EMG hybrid energy-harvesting shock absorber adapted from [81].

Yang et al. [78] targeted linear suspension vibration using a retractable spring-like electrode TENG (SL-TENG) in which spring steel served simultaneously as the structural framework and electrode, as shown in Figure 3a. The flexible three-layer architecture used the available installation space more efficiently and promoted synchronized contact–separation motion. A circular central opening allowed the device to be mounted around the suspension support rod, so the SL-TENG followed spring motion while harvesting vibration energy. Kang et al. [79] addressed reciprocating shock-absorber motion with a cylindrical suspension-type freestanding-mode triboelectric generator (STEG). Figure 3b shows different triboelectric materials patterned as gratings on the inner and outer cylinders of the shock absorber. Relative sliding between the cylinders produces friction between the perfluoroalkoxy (PFA) film on the inner cylinder and the opposing surface, thereby generating triboelectric charge.

For sensing, suspension motion is an important input to whole-vehicle dynamics control, and its response to uneven roads directly determines the triboelectric waveform generated by a TENG. Analyzing these signals can provide information on road roughness, suspension travel, and vehicle load variation in real time without external power or complex wiring. Yan et al. [80] developed a triboelectric dual-parameter sensor (TDS) based on vehicle–road coupling for integration into a suspension shock absorber. Figure 3c presents the device structure and operating principle. A triboelectric displacement sensing unit (TDSU) using a linear guide–slider mechanism measured vertical suspension displacement, whereas a pendulum-based triboelectric inclination sensing unit (TISU) measured the road slope angle. Combining the two measurements enabled an intelligent visualization system for road parameters. Hu et al. [81] integrated a TENG with an electromagnetic generator (EMG) to recover suspension vibration energy. The hybrid generator shown in Figure 3d was embedded directly in the shock absorber. Microstructured polyimide (PI) films increased TENG durability, while a Halbach magnet array improved EMG output. Because the hybrid generator moved synchronously with the original shock absorber, it could enable self-powered operation of an accelerometer without appreciably changing the suspension’s dynamic response.

### 3.3. Collision Monitoring

Changes in vehicle body vibration amplitude, frequency, and multi-axis acceleration are closely associated with equipment operating conditions, road irregularity severity, and vehicle attitude. Reliable acquisition of these vibration waveforms can therefore provide a broad set of indicators for evaluating vehicle condition.

**Figure 4:**
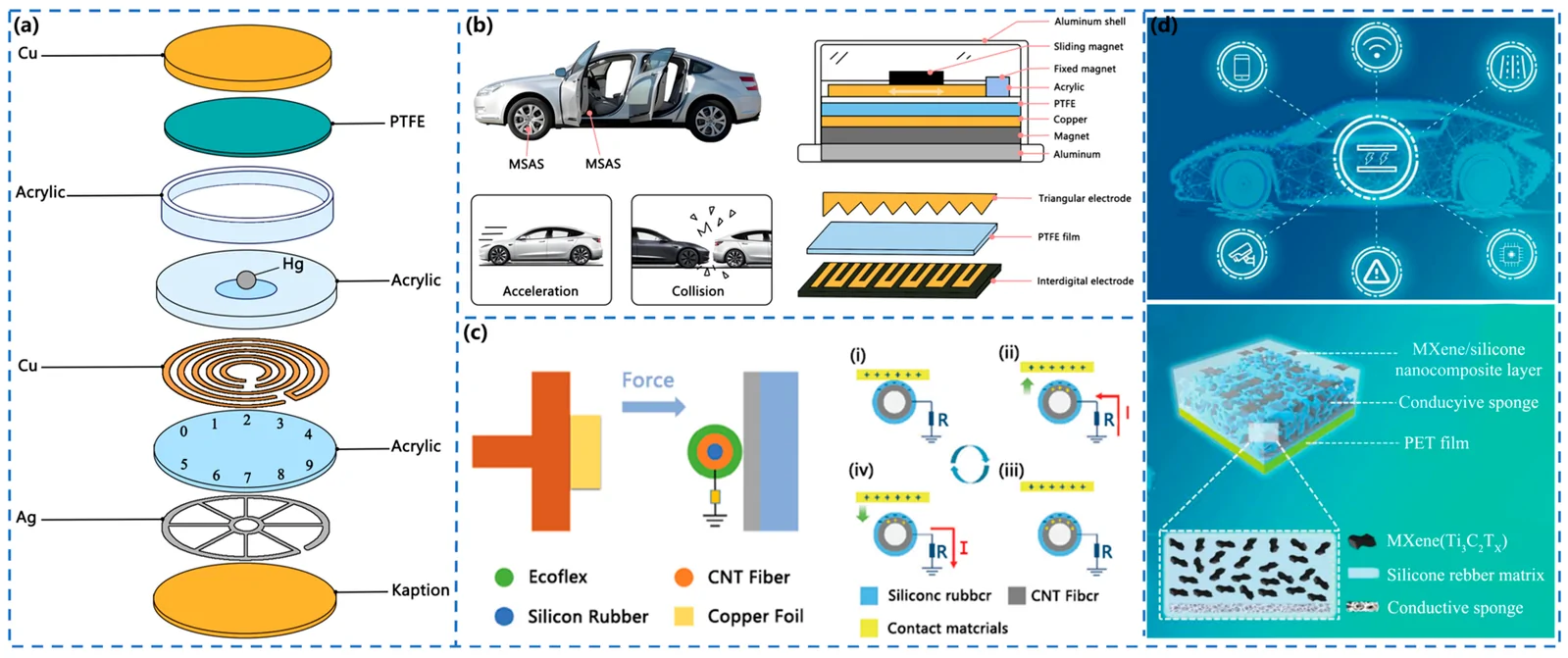
Triboelectric sensors for vehicle collision monitoring and noncontact early warning. (**a**) Coded liquid metal TENG-based three-dimensional acceleration sensor adapted from [82]. (**b**) Magnetically assisted self-powered acceleration sensor (MSAS) and collision monitoring scenario adapted from [83]. (**c**) Approach/withdrawal sensing with a mechanoluminescent triboelectric nanogenerator fiber (MLTENGF) [84]. (**d**) MXene/silicone-nanocomposite-enhanced self-powered noncontact triboelectric nanogenerator (SNC-TENG) for intelligent vehicle perception [85].

TENG-based self-powered sensing has also been applied to collision monitoring. Zhang et al. [82] used a liquid metal triboelectric nanogenerator (LM-TENG) to construct the lightweight, low-cost, highly sensitive three-dimensional acceleration sensor shown in Figure 4a. Vehicle motion caused the mercury droplet to roll inside the device, and abrupt changes in droplet position were used to locate a collision. Lu et al. [83] extended the measurable acceleration range with a magnetically assisted self-powered acceleration sensor (MSAS) based on a TENG. The device covered the low accelerations of normal driving as well as the much higher accelerations associated with collisions. As illustrated in Figure 4b, acceleration was derived from the frequency of current pulses produced when the sliding magnet contacted the interdigital electrodes.

TENG research has progressed from post-collision detection toward noncontact collision warning. Wu et al. [84] produced a stretchable self-powered mechanoluminescent triboelectric nanogenerator fiber (MLTENGF) from lightweight carbon nanotube fibers using a customized combination of winding and three-dimensional coating. The operating mechanism is illustrated in Figure 4c. With copper foil representing a vehicle, the direction of MLTENGF-induced current distinguished approach from withdrawal. Zhao et al. [85] subsequently extended the noncontact detection range using a self-powered noncontact triboelectric nanogenerator (SNC-TENG), whose application and working mechanism are shown in Figure 4d. MXene (Ti_3_C_2_Tₓ) nanosheets together with a mesh-structured, fully embedded conductive sponge increased the triboelectric charge density. This configuration enabled recognition of human activity within 2 m and detection of displacements down to 1 mm. Relative to previously reported noncontact TENGs, the SNC-TENG also exhibited a comparatively large unit-area perception distance.

## 4. Wheel–Road Systems

The wheel–road system directly couples the vehicle to the road and is therefore central to both mechanical energy dissipation and vehicle–road information exchange. This interface contains substantial amounts of otherwise wasted recoverable mechanical energy together with valuable dynamic information on road conditions. TENGs technology harvests energy and performs condition sensing at wheel-end components, including tires, bearings, and braking components, while also supplying information on road state and traffic flow.

### 4.1. Wheel Compression

**Figure 5:**
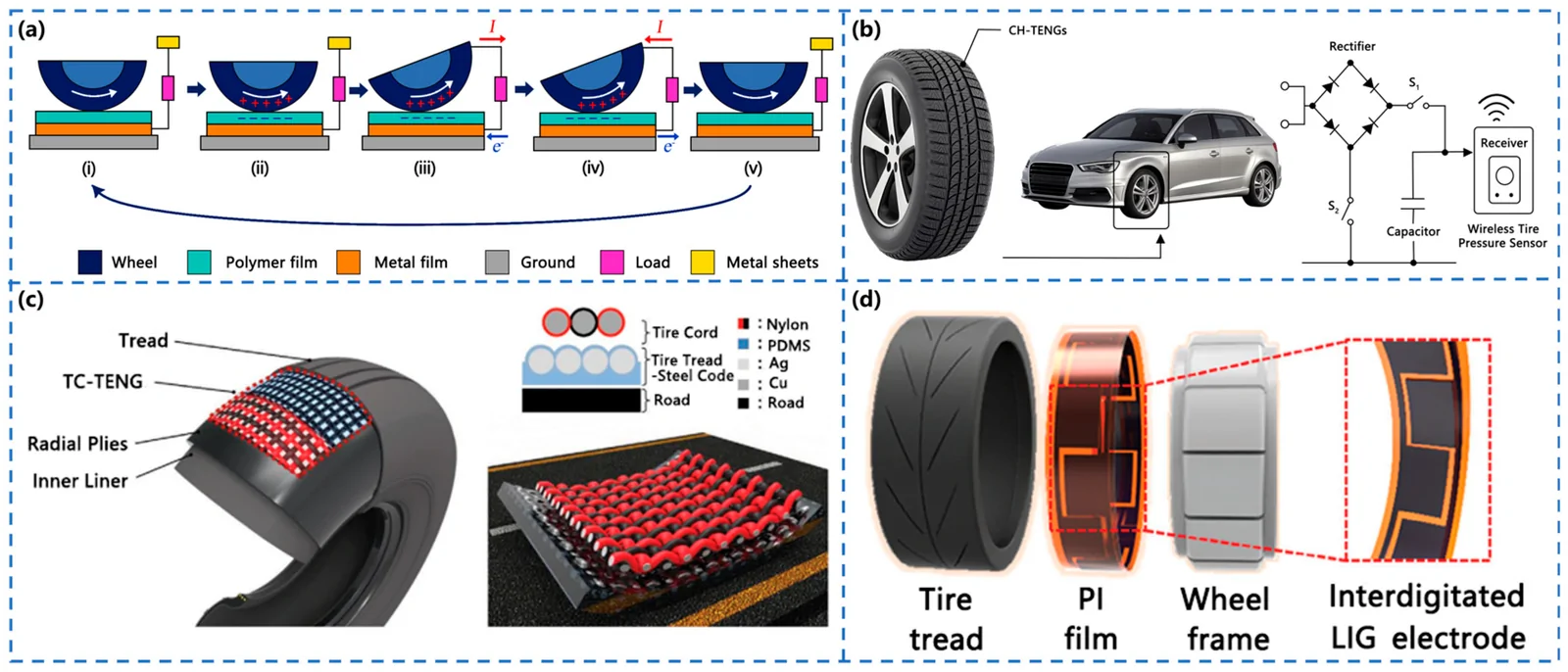
Triboelectric energy harvesting and condition sensing under wheel compression and contact. (**a**) Single-electrode TENG (S-TENG) for harvesting frictional energy from rolling tires adapted from [86]. (**b**) Compressible hexagonal-structured TENG (CH-TENGs) array used to power a wireless tire pressure sensor adapted from [87]. (**c**) Dual-friction-mode textile tire cord TENG [88]. (**d**) Real-time tire monitoring system based on interdigitated electrodes and hybrid deep learning [91].

Continuous wheel–road interaction is accompanied by substantial mechanical energy transfer and dissipation. Because the tire and road are dissimilar contacting materials, their interface can be used directly for triboelectric energy harvesting, with the two surfaces acting as charge-generating materials. Mao et al. [86] developed a single-electrode triboelectric nanogenerator (S-TENG) in which a rough polydimethylsiloxane (PDMS) film represented the tire surface. Figure 5a presents the device structure and working principle. Contact between the PDMS film and the ground transferred electrons from the ground to the PDMS. Repeated contact and compression during tire rolling then enabled the S-TENG to recover frictional energy from the wheel–road interface.

To enlarge the effective contact area, Guo et al. [87] arranged compressible hexagonally-structured triboelectric nanogenerators (CH-TENGs) into the array shown in Figure 5b. Parallel stacking allowed multiple CH-TENG units to be fixed inside a rubber tire, where the mechanically stable structure could operate continuously while powering a wireless tire pressure sensor. This integration strategy nevertheless replaces part of the region between the tire tread and inner liner with an elastic material and may compromise tire reliability. Seung et al. [88] pursued a more fully integrated tire-compatible design by fabricating the TENG from textile-based tire materials, as shown in Figure 5c. Polydimethylsiloxane (PDMS)-coated silver textile formed the outer tread material, while woven nylon served as the inner tire-cord material. The resulting architecture provided two frictional contacts without sacrificing a flat tread surface.

The electrical signals produced by TENGs during tire motion also enable self-powered sensing. Xu et al. [89] developed a sandwich-type triboelectric nanogenerator (St-TENG) to estimate tire load. Its multilayer configuration maintained close conformal interfacial contact while preventing contaminant ingress that could disturb electrical output. The St-TENG acquired tire carcass deformation in real time and used the corresponding electrical response to estimate tire load and other key parameters accurately.

For compression-based sensing, Pang et al. [90] developed a textile-inspired intelligent tire TENG that combined energy harvesting with self-powered monitoring of pavement skid resistance. A lightweight algorithm processed the real-time TENG output to quantify skid resistance. Kim et al. [91] subsequently reported an environmentally robust real-time tire monitoring platform based on TENG sensing that identified road markings and road surface conditions. During rolling contact, the interdigitated electrodes shown in Figure 5d generated continuous triboelectric waveforms. A hybrid deep-learning approach based on time–frequency representations was then used to extract driving information from these signals.

### 4.2. Wheel Rotation

**Figure 6:**
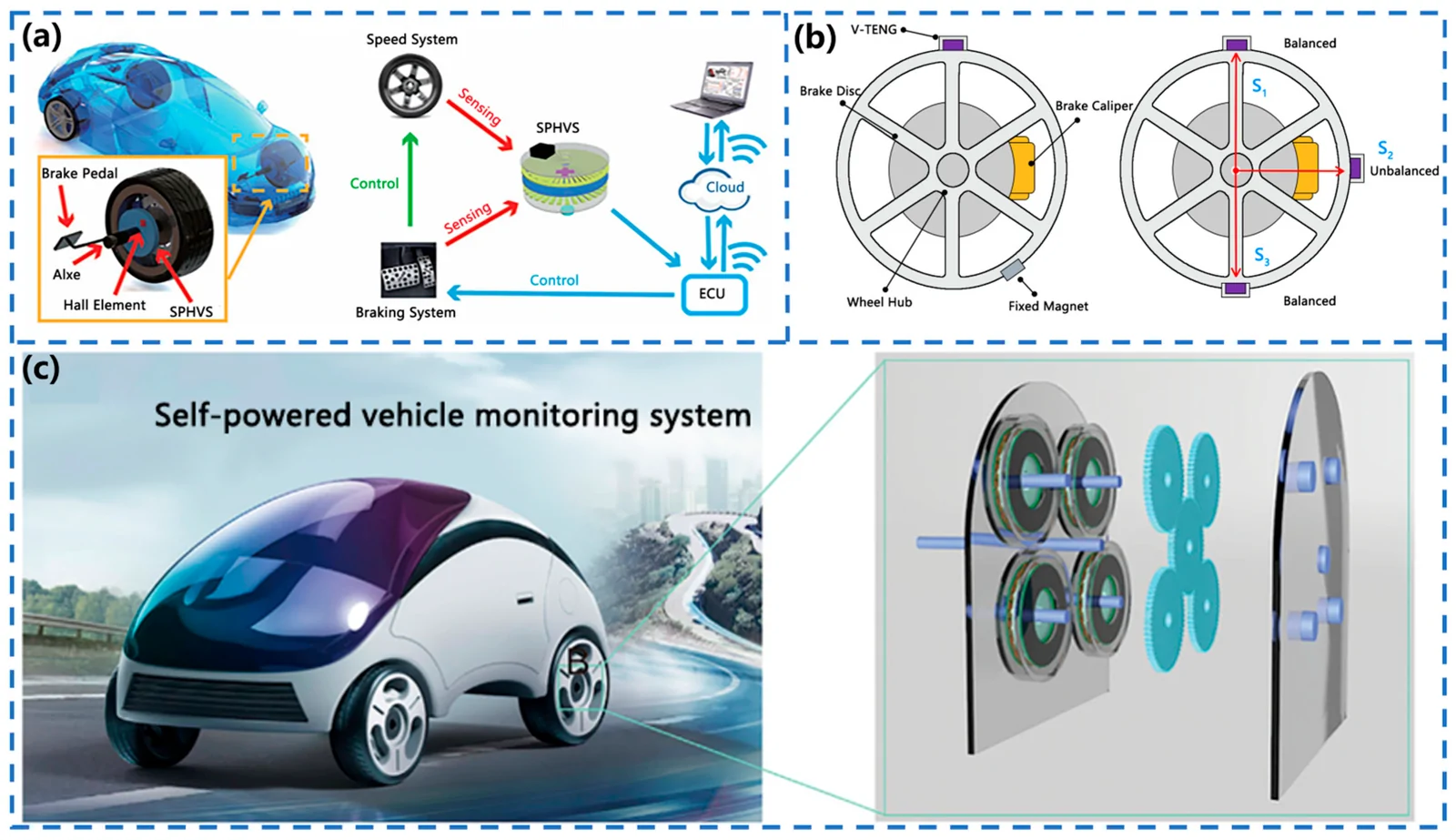
Rotational energy harvesting from wheels and bearings and self-powered vehicle monitoring. (**a**) Disk-type TENG-based self-powered Hall vehicle sensor (SPHVS) and automotive safety monitoring system [93]. (**b**) Wheel-hub installation, balance structure, and tire pressure monitoring of the on-vehicle magnetically actuated TENG (V-TENG) adapted from [94]. (**c**) 3D-printed bearing-structured TENG (BS-TENG) and self-powered vehicle monitoring system [95].

Rotational wheel motion is another important source of both mechanical energy and sensing information. Li et al. [92] recovered this energy with a freestanding rolling-mode triboelectric nanogenerator (FR-TENG) composed of a 3D-printed tire, internal structural layers, and rolling elements. Three rolling-element geometries were compared, and the cylindrical element provided more stable motion together with improved electrical output. Guo et al. [93] then coupled wheel rotation harvesting to a self-powered Hall vehicle sensor (SPHVS). A disk-type TENG supplied stable power to the Hall element over the tested rotational speed range, and the SPHVS-based automotive safety monitoring system in Figure 6a used overspeed information to support serious traffic accident prevention. Qian et al. [94] developed an on-vehicle magnetically actuated triboelectric nanogenerator (V-TENG) that could be installed directly on the wheel hub within a pneumatic tire (Figure 6b). Its seesaw-balance architecture counteracted centrifugal force at high speed and increased effective contact area by a factor of four. As a result, the V-TENG maintained high output over a broad rotational speed range and could supply a battery-free tire pressure sensor.

Bearing rotation provides another localized mechanical energy source. Yang et al. [95] fabricated a bearing-structured triboelectric nanogenerator (BS-TENG) by 3D printing and configured it to rotate synchronously with the main shaft, as shown in Figure 6c. The authors combined eight BS-TENG units into a self-powered vehicle monitoring (SPVM) system. The resulting system recovered rotational energy and simultaneously measured wheel temperature and rotational speed.

### 4.3. Intelligent Road and Traffic Monitoring

The preceding studies recover mechanical energy mainly by placing TENGs in wheel-end components exposed to tire deformation or rotation. At the vehicle–road coordination level, a complementary strategy is to integrate triboelectric sensing directly into the road infrastructure: TENGs embedded at the pavement surface can directly acquire mechanical excitation produced by vehicle–road contact.

**Figure 7:**
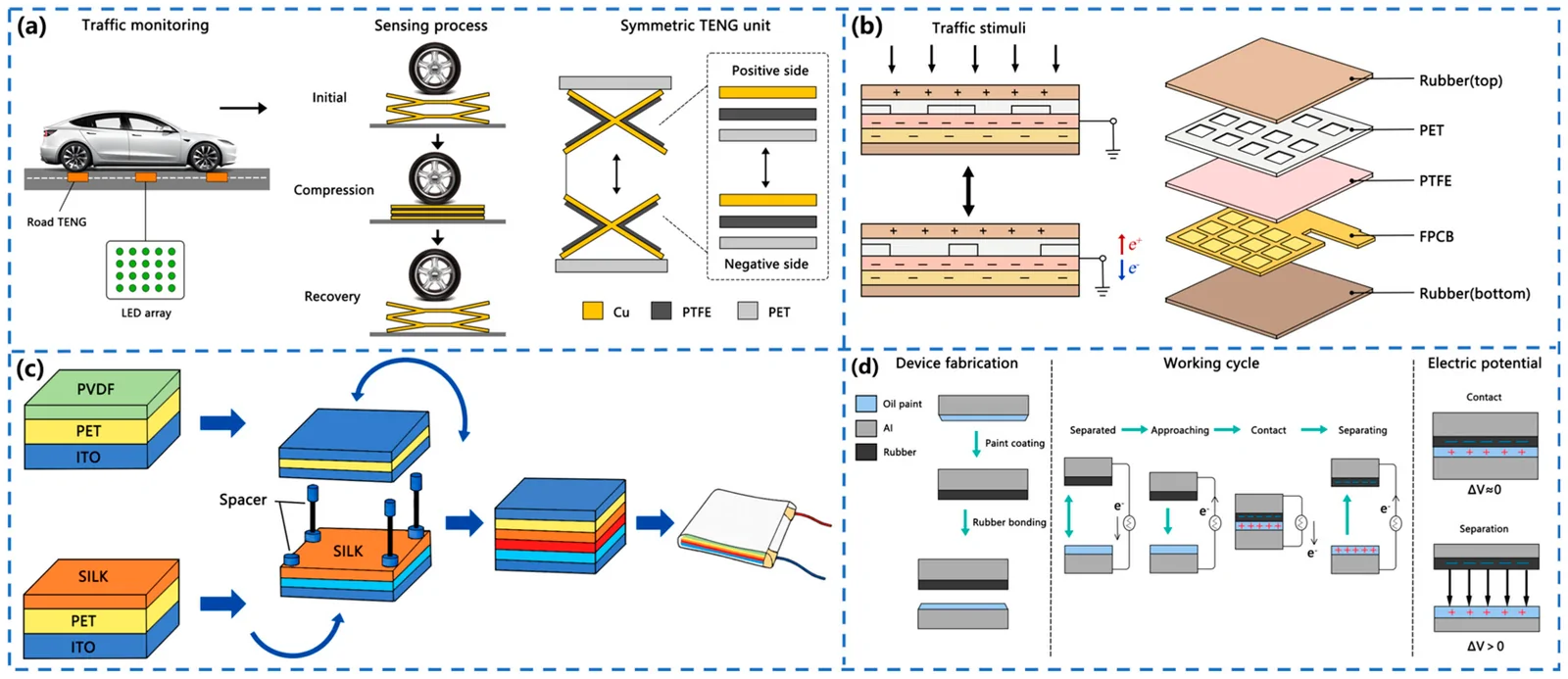
Triboelectric nanogenerators for smart roads and traffic monitoring. (**a**) Waterbomb-origami-inspired TENG (WO-TENG) and traffic monitoring principle adapted from [96]. (**b**) Self-powered smart transportation infrastructure skin (SSTIS) and vehicle axle load identification adapted from [97]. (**c**) Silk–PVDF TENG (SP-TENG) structure and vehicle load monitoring adapted from [98]. (**d**) Material structure and electromechanical response of the road-compatible TENG (r-TENG) adapted from [99].

Pang et al. [96] developed a waterbomb-origami-inspired triboelectric nanogenerator (WO-TENG) that was encapsulated and embedded in pavement to form a traffic monitoring sensor system, as shown in Figure 7a. Two independent waveform features, voltage amplitude and time interval, were analyzed to accurately determine vehicle speed, traffic volume, and vehicle type. For more detailed vehicle characterization, Zheng et al. [97] attached thin, flexible bioinspired TENG sensors to the pavement to create a self-powered smart transportation infrastructure skin (SSTIS). Figure 7b shows its structure and operating mechanism. Mechanical excitation from a passing vehicle drove vertical contact–separation in the TENGs and generated electrical signals. A deep-learning algorithm processed these signals to identify axle load characteristics among different vehicles. Each TENG sensor set contained eight sensing units spanning the full vehicle passage event and achieved an overall vehicle classification accuracy of 81.06%.

For macroscopic monitoring of traffic conditions and vehicle loads, Sarkar et al. [98] fabricated a flexible triboelectric nanogenerator prototype (SP-TENG) based on a silk–polyvinylidene fluoride (PVDF) triboelectric pair. The device counted passing vehicles and monitored traffic congestion; Figure 7c shows its structure and operating principle. Pulse amplitude from the SP-TENG was used to determine passing vehicle weight, enabling an automated pavement-based platform for overload detection. TENG sensing can additionally be extended from vehicle characteristic recognition to trajectory analysis. Fu et al. [99] developed a road-compatible triboelectric nanogenerator (r-TENG), shown in Figure 7d, whose signal variations indicated events such as crossing lane markings or deviating from an intended route. Intelligent algorithms could further process these signals to analyze driver behavior and identify lane line crossings or route deviations. The resulting information can provide real-time feedback to help trainee drivers recognize and correct training errors.

For system-level integration and active safety, Wang et al. [100] embedded a TENG in a road speed bump to detect overloaded vehicles, recognize roadway behavior, and activate traffic warnings. Mechanical input from passing vehicles was converted into electrical energy. The generated electricity supported basic monitoring functions and could also trigger warning alarms, thereby improving road safety.

## 5. Intelligent Vehicle Systems

Vehicle cabins and other onboard subsystems contain numerous micromotion sources during normal operation. Integrating TENGs at these locations can both recover local mechanical energy and enable multisource condition sensing. Research on intelligent vehicle systems increasingly combines information sensing with human–machine interaction, with particular emphasis on extracting high-value information from low-energy environments. Accordingly, TENG-based smart cockpit interfaces, human–machine interaction devices, and vehicle fluid monitors have become active research areas.

### 5.1. Smart Cockpit

**Figure 8:**
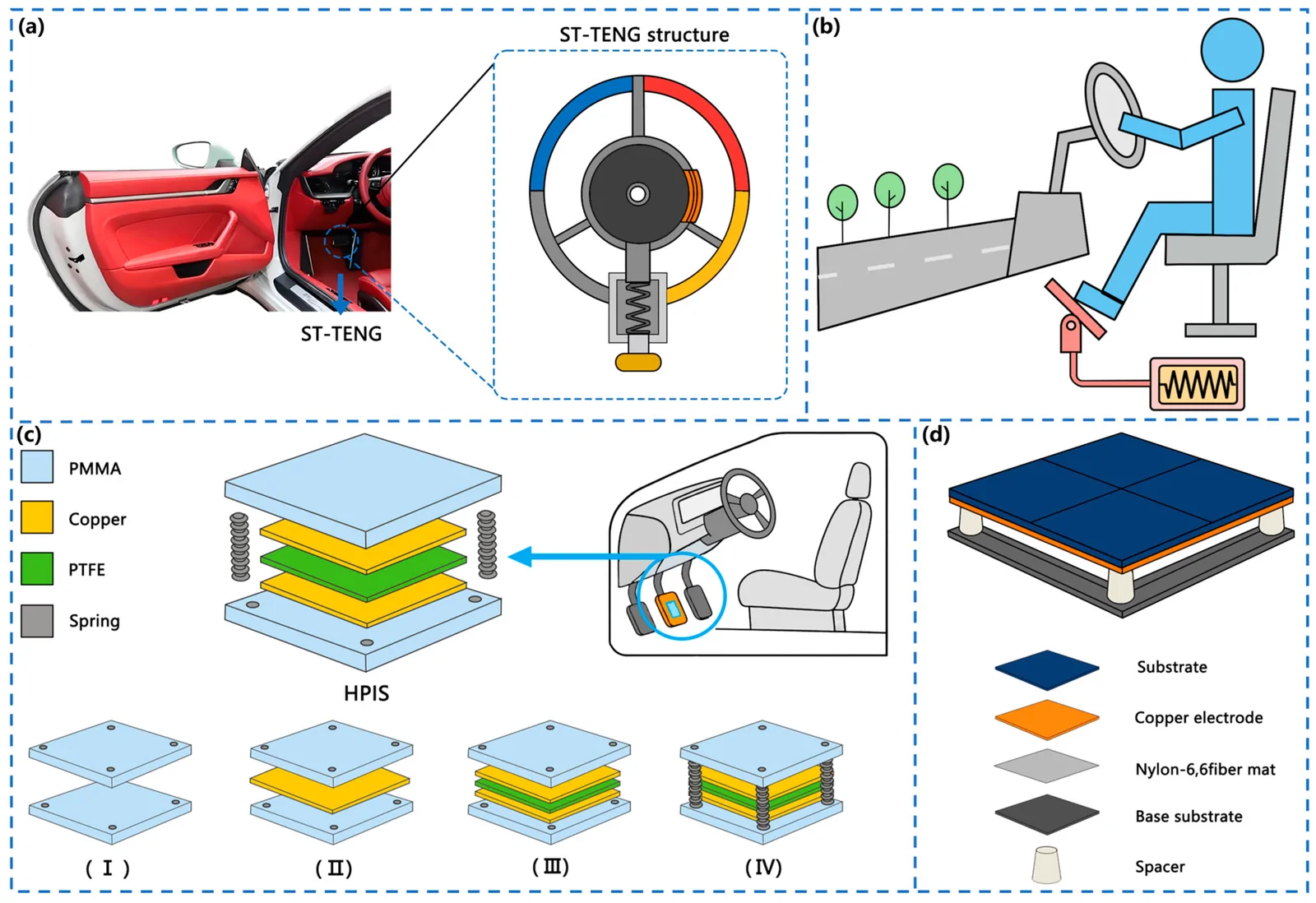
Triboelectric energy harvesting and driver state monitoring in smart cockpits. (**a**) Footwell/pedal installation, device structure, and driving habit monitoring of the sweep-type TENG (ST-TENG) adapted from [101]. (**b**) Brake pedal integration of the gradient and multimodal TENG (GM-TENG) adapted from [102]. (**c**) Human–pedal interaction sensor (HPIS) for longitudinal driving intention recognition adapted from [103]. (**d**) Nylon-6,6-nanofiber TENG for seat occupancy monitoring adapted from [104].

Cockpit controls such as steering wheels and brake pedals continuously produce subtle mechanical inputs. Because TENGs are readily integrated and operate without an external power source, they can capture these control motions for passive real-time monitoring of steering and braking states. Xie et al. [101] developed a sweep-type triboelectric nanogenerator (ST-TENG) for harvesting randomly triggered cockpit motion. The device shown in Figure 8a comprised a push rod, housing, two flywheels, and one freewheel, and its electrical output reflected the characteristics of the triggering motion. Pedal actuation signals could therefore be extracted to characterize differences in driving habits among drivers. Pang et al. [102] addressed brake pedal sensing and road surface feedback using a gradient and multimodal triboelectric nanogenerator (GM-TENG) inspired by naturally occurring triply periodic minimal surface (TPMS) structures. The device provided high sensitivity and multimodal sensing for driving safety monitoring and was installed beneath the brake pedal after encapsulation, as shown in Figure 8b. Variations in its output provided information on pavement skid resistance. To recognize longitudinal driving intent earlier from pedal movement, Tan et al. [103] developed a hybrid data- and model-driven system for early recognition of fine-grained longitudinal driving intentions (LDIPRS). A TENG-based human–pedal interaction sensor (HPIS) monitored fine-grained longitudinal maneuvers. Figure 8c presents the spring-supported vertical contact–separation structure of the HPIS. Triboelectric signals generated before the vehicle response were then used to monitor driver foot movement.

Beyond cockpit-control sensing, vehicle seats provide another important location for monitoring occupant status. Mathew et al. [104] developed a surface-area-enhanced TENG from neat electrospun Nylon-6,6 nanofibers for vibration energy harvesting and seat occupancy detection. Figure 8d shows a device composed of four functional blocks. An acrylic foam substrate maintained seating comfort while protecting the structure, and customized rubber spacers increased device durability. The resulting TENG identified whether the seat was occupied and provided a corresponding visual indication.

Xu et al. [105] developed a real-time driver state monitoring and intelligent fatigue warning system based on steering behavior. The system combined a TENG-based self-powered steering wheel angle sensor (SSAS) with a signal-processing unit. Mounted directly on the steering shaft, the SSAS measured steering wheel angle at a resolution of 1°. The processing unit analyzed the angle data to extract characteristic parameters indicative of driver state, enabling continuous monitoring.

Current research is also moving from single-variable sensing toward system-level multisensor fusion. Zhang et al. [106] developed a driver training assistance system (DTAS) that acquired a broader set of driving signals through gear shift, steering angle, and pedal sensors. During gear shifting, the gear lever drove an acrylic slider and produced sliding friction. Turning the steering wheel rotated the rotor relative to the stator, whereas pedal actuation repeatedly brought components into contact and separation. Charge transfer generated by these three processes enabled integrated monitoring of driving behavior.

### 5.2. Human–Machine Interaction

**Figure 9:**
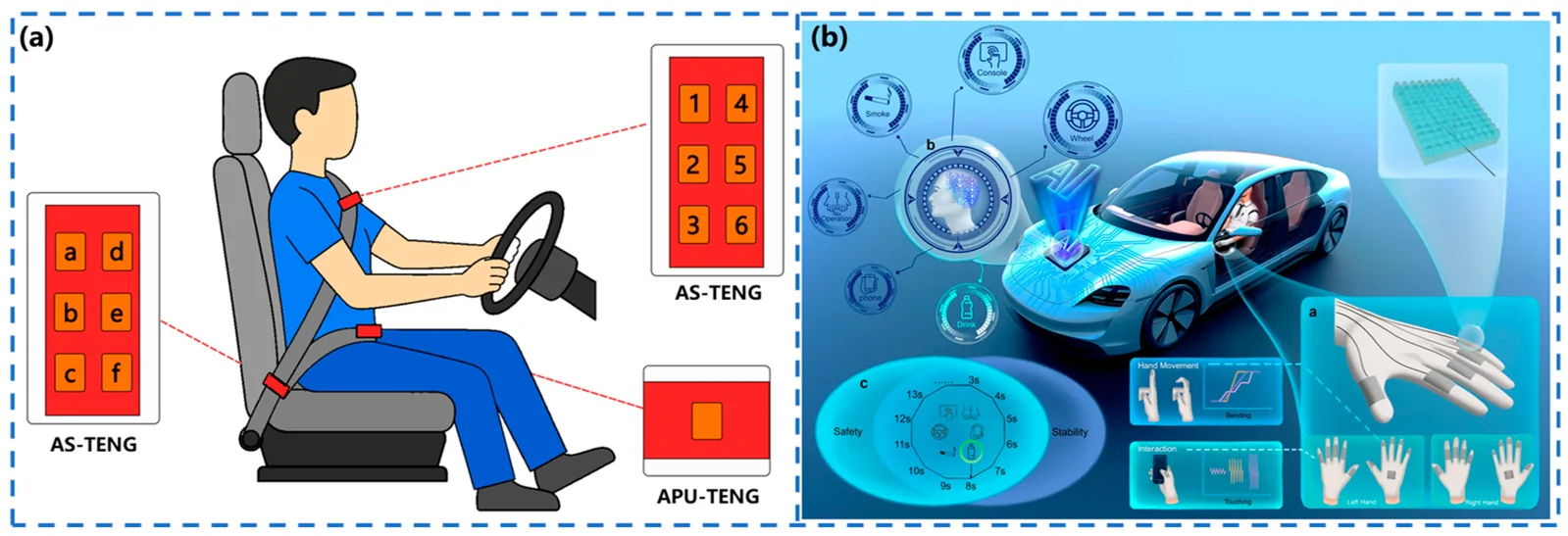
Triboelectric sensing for driver status monitoring and takeover assistance in automated driving. (**a**) Self-powered smart safety belt integrating APU-TENG and AS-TENG arrays for driver status monitoring adapted from [107]. (**b**) Driving behavior recognition and takeover-time adjustment based on triboelectric sensor gloves [108].

Accurate recognition of driving behavior is essential for automotive safety. Flexible TENGs incorporated into seat belts and smart gloves can convert human motion into triboelectric electrical signals for human–vehicle interaction and passive detection of unsafe actions. Feng et al. [107] implemented this concept in a self-powered smart safety belt that used two TENG types to monitor forward driver displacement and turning actions; Figure 9a shows the application and structural layout. An auxetic polyurethane triboelectric nanogenerator (APU-TENG) embedded in the horizontal strap sensed forward displacement with a strain sensitivity of 0.89 V cm^−2^ and captured a broad range of driver displacements during emergency deceleration. The diagonal strap carried two sensor arrays at the shoulder and waist. Each array contained six arch-shaped TENG (AS-TENG) units, enabling identification of turning direction and turning angle, respectively. This design integrates triboelectric motion sensing into flexible components that interact directly with the human body and broadens the application prospects of TENG-based wearable sensors in automotive applications.

Automated-driving technologies are advancing rapidly, particularly in new energy vehicles, yet driver takeover remains a critical constraint on their wider deployment and further development. Lu et al. [108] addressed this issue with the intelligent takeover assistance system (ITAS) shown in Figure 9b. The system used all-round sensing gloves (AS-Gloves) equipped with triboelectric sensors to capture subtle hand movement and hand–object interaction without interfering with the driver. These behavioral inputs were converted into electrical signals and transmitted to a non-driving behavior identification module. A deep-learning real-time recognition module then identified non-driving behavior and provided the information required for the driver to resume control safely. The authors also developed a takeover recovery time reasoning system to infer takeover recovery time directly and in real time, addressing the need for dynamic calibration of autonomous driving takeover time. Together, these functions help ensure the safety and stability of the takeover process while accommodating complex real-world driving scenarios.

### 5.3. Vehicle Fluid Monitoring

**Figure 10:**
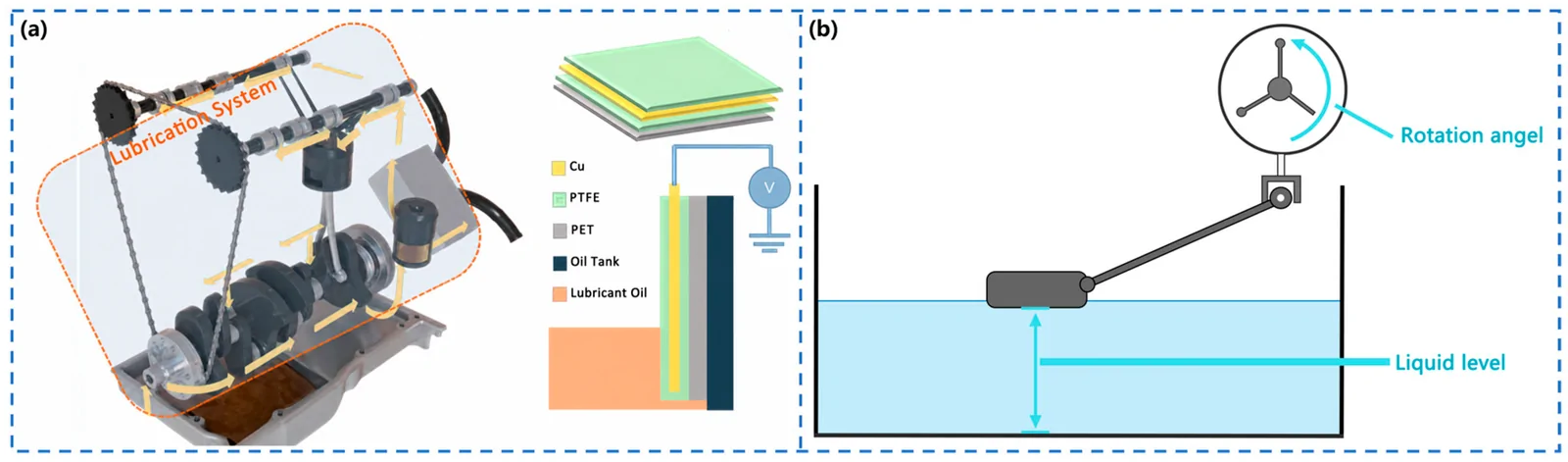
Triboelectric monitoring of vehicle lubricating oil condition and fuel level. (**a**) Oil–solid contact-electrification TENG (O–S TENG) for lubricating oil condition monitoring and device structure [109]. (**b**) Fuel level measurement principle of the dual-mode triboelectric liquid-level monitoring system (TLLMS) adapted from [110].

Vehicle fluid condition and level are closely related to driving safety, energy efficiency, and maintenance requirements. TENG devices can exploit vehicle vibration and fluid motion as mechanical inputs and therefore support continuous low-power monitoring of lubricant condition and fuel level. Zhao et al. [109] applied an oil–solid contact-electrification triboelectric nanogenerator (O–S TENG) as a self-powered sensor for real-time lubricating oil assessment; Figure 10a shows the application and device structure. Wear debris, carbon deposits, and aged organic compounds change charge transfer at the liquid–solid interface, allowing for the detection of common contaminants in base oil through variations in the electrical response. The device is powered by vibrational energy generated by the vehicle’s acceleration and deceleration, which enables continuous signal collection. Li et al. [110] studied the fuel level assessment using a low-power, high-precision triboelectric liquid-level monitoring system (TLLMS) as shown in Figure 10b. A fluctuating liquid level made a float rotate the primary shaft of the triboelectric liquid-level sensor (TLLS). The height of the liquid level was instantaneously computed according to the related rotational angle. Two triboelectric modes offered complementary encoding capabilities, with a freestanding triboelectric nanogenerator (FS-TENG) serving as an incremental encoder and a single-electrode triboelectric nanogenerator (SE-TENG) serving as an absolute encoder. A moving-average algorithm filtered disturbances from fuel-tank vibrations during vehicle operation, giving high measurement stability. The system’s average power consumption was approximately 24.7% of that of commercial sensors.

## 6. Conclusions and Future Perspectives

### 6.1. Conclusions

This review systematically discusses the recent advances in triboelectric nanogenerators for automotive energy harvesting and intelligent sensing and their promising potential to boost smart vehicle technologies. The main studies are grouped into three application areas: vehicle vibration, wheel–road, and intelligent vehicle systems. A considerable amount of literature is devoted to simultaneous energy harvesting and sensing, which is in line with the intrinsic relationship between energy conversion and signal sensing in TENGs. The review therefore classifies applications by vehicle systems while discussing the two functions in parallel. Table 1 and Table 2 consolidate the key parameters and essential information on TENG implementations across the two application functions.

In vehicle vibration systems, mechanical energy is recovered from body and suspension motion, and the associated triboelectric signals are used to evaluate operating conditions and support functions such as collision warning. At the wheel–road interface, energy arising from wheel compression and rotation can likewise be harvested, while TENG-based monitors embedded in tires or pavement provide information on traffic flow, vehicle load, and road conditions. Research on intelligent vehicle systems extends sensing to information generated both inside and outside the vehicle, including cockpit operation, human–machine interaction, and vehicle fluid status. These applications provide sensing capabilities relevant to intelligent driving. These developments may improve vehicle energy efficiency and facilitate distributed sensing networks for intelligent vehicles, but important limitations remain. Present TENGs still exhibit insufficient energy conversion efficiency and power supply capacity for continuous and stable operation of onboard sensors. Most intelligent sensing implementations also monitor only a single parameter, and both measurement accuracy and the range of detectable variables require improvement. At the system level, existing studies primarily integrate TENGs into individual vehicle components; deeper integration with onboard energy and electronic control systems has not yet been achieved.

### 6.2. Future Perspectives

This review summarizes the state of the art in TENG research for vehicle energy harvesting and intelligent sensing, highlights the technical challenges for broader implementation in vehicles, and proposes possible development directions.

The output performance of TENGs for energy harvesting still needs to be improved. Future work could focus on the design of generators better adapted to vehicle operating conditions, dielectrics with better durability through material selection or modifications, and optimized energy management circuits. Cognitive sensing requires concurrent improvements in precision and reliability. Multimodal TENG designs should be diversified to expand the types of measurable variables. Extensive experimental and simulation datasets are necessary for the improvement of signal decoding and parameter estimation algorithms. Effective in-vehicle integration is also important for practical deployment. TENG devices should be embedded in existing vehicle components, be compatible with and not interfere with electronic control or motion systems, and be progressively adapted to vehicle-grade standards. The continuous progress and practical utilization of vehicle TENGs promote vehicle energy efficiency and intelligence while offering new technological support for intelligent transportation.

## Figures and Tables

**Table 1:** Summary of representative TENGs for vehicle energy harvesting.

Device	Mode	Mounting Position	Output Performance	Key Test Conditions	Applications	References
EV-TENG	Vertical contact–separation mode	Automobile engine	Open-circuit voltage: ≈75 V; peak short-circuit current: 10 μA	10–30 Hz (600–1800 rpm)	Detect automobile toxic exhaust gases with a WO_3_-based NO_2_ chemoresistive gas sensor	[71]
DIV-TENG	Vertical contact–separation mode	Front dashboard and rear deck	Peak power: 0.075 mW (10 MΩ)	Low-frequency characterization range: 2–5 Hz	Harvest broadband vehicle vibration energy to power LED indicator arrays	[72]
SL-TENG	Vertical contact–separation mode	External brace mount	Peak voltage: 80 V Peak current: 14 μA	10 Hz; amplitude: 5 mm	Self-powered vibration sensor to monitor road potholes	[78]
STEG	Freestanding triboelectric-layer mode	—	RMS output voltage: 131.9 V	Reciprocating speed: 200 mm s^−1^; displacement: 50 mm; damping force: 0.6 kgf	Supply power for vehicle low-power ADAS sensors	[79]
CH-TENGs	Vertical contact–separation mode	Fixed in a rubber tire	Peak power: 1.9 mW (8 units, 65 MΩ)	Tire speed: 2.51 m s^−1^; normal load: 10 N	Supply power to commercial wireless tire pressure sensor by harvesting tire rotation energy	[87]
TC-TENG	Vertical contact–separation mode	Inside the tire	Maximum output power: ≈0.5 mW (four units, 10 MΩ)	Rotational speed: 1000 rpm	Operate wireless speed sensor and miniature power-consuming electronics	[88]
V-TENG	Vertical contact–separation mode	Wheel hub	Maximum power density: 16.44 W/m^2^ (3 MΩ)	Rotational speed: 100 rpm; magnetic field: 0.2 T	Power low-power wireless sensors and miniature electronic devices	[94]
BS-TENG	Rolling mode	Mounted on the main shaft	Peak power: 0.96 mW (one unit, 8 MΩ)	Rotational speed: 600 rpm	Harvest vehicle rotational energy for self-powered speed and temperature monitoring sensors	[95]
ST-TENG	Rolling mode	Mounted under the pedals	Maximum output power: 2.4 mW (two generating units in parallel, 70 MΩ)	Triggering speed: 1.2 m s^−1^	Power temperature–humidity sensor and monitor driving habits	[101]

The reported output values were obtained under different excitation conditions, device dimensions, electrical loads, and device configurations and are therefore not directly comparable across studies.

**Table 2:** Summary of representative TENGs for vehicle intelligent sensing.

Device	Mode	Information	Sensing Range	Sensitivity	Applications	References
VS-TENG	Vertical contact–separation mode	Mounted on compressor top, detects vibration signals of 1–2000 Hz	Vibration frequency: 1–2000 Hz	—	Machinery condition monitoring	[77]
TDS	Freestanding triboelectric-layer and single-electrode modes	Integrated into vehicle suspension shock absorbers, measures pavement roughness and road slope angle	Displacement: 5–80 mm; slope angle: 6–30°	—	Intelligent road parameter visual characterization system	[80]
SNC-TENG	Noncontact single-electrode mode	Vehicle-surface-mounted, realizes ultralong-distance signal detection	Human-activity detection distance: up to 2 m	Minimum detectable movement: 1 mm	Vehicle security and collision detection	[85]
St-TENG	Vertical contact–separation mode	Mounted on tire inner surface, collects real-time tire-load information	Tested tire load: 4000–12,000 N	—	Tire-load and tire-parameter monitoring	[89]
SPHVS	Freestanding triboelectric-layer mode	Fixed on the vehicle’s front left wheel, monitors vehicle speed and braking signals	Vehicle speed: 4.7–122.0 km h^−1^; brake pedal angle: 10–70°	0.22 Hz h km^−1^ (vehicle speed); 0.08 mV °^−1^ (brake pedal angle)	Automobile safety monitoring	[93]
SSTIS	Single-electrode mode	Attached to road surface, identifies vehicle type and axle load data	Four tested total weight classes: 24,663.3, 27,563.3, 29,745.0, and 32,556.7 kg	—	Traffic and vehicle axle load classification	[97]
SP-TENG	Vertical contact–separation mode	Arranged on road surface under tires, detects overload information and vehicle count	Tested applied load: 20–160 kg	—	Automotive toll-plaza tax verification	[98]
GM-TENG	Vertical contact–separation mode	Installed between the brake pedal and vehicle bottom plate, monitors speed, load, and working mode	Applied load: 5–260 N	0.14, 0.04, and 0.11 N V^−1^ (Modes 1–3, respectively)	Driving safety monitoring in intelligent transportation system	[102]
AS-Gloves	Single-electrode mode	Placed on the finger knuckles, detects delicate hand motions and hand-object interaction	Applied force: 0–6 N	3.257 V N^−1^ (0–3.5 N); 0.706 V N^−1^ (3.5–6 N)	Takeover-time adjustment in conditionally automated vehicles	[108]
O−S TENG	Single-electrode mode	Attached to the inner wall of the tank, monitors lubricating oil condition and contamination	Fe debris/carbon black: 0–20 mg mL^−1^; diesel oil: 0–30 wt%; water: 0–1 wt%	Demonstrated detectable levels: 1 mg mL^−1^ debris; 0.01 wt% water	Real-time engine-oil monitoring and intelligent diagnosis	[109]

The reported sensing ranges, sensitivities, and detection capabilities were obtained for different sensing targets under different device configurations, test conditions, and evaluation methods and are therefore not directly comparable across studies.

## Data Availability

No new data were created or analyzed in this study. Data sharing is not applicable to this article.
